# A Chemical Biology Approach to Probing the Folding Pathways of the Inhibitory Cystine Knot (ICK) Peptide ProTx-II

**DOI:** 10.3389/fchem.2020.00228

**Published:** 2020-04-03

**Authors:** Stephen McCarthy, Jenna Robinson, Konstantinos Thalassinos, Alethea B. Tabor

**Affiliations:** ^1^Department of Chemistry, UCL, London, United Kingdom; ^2^Department of Biological Sciences, Institute of Structural and Molecular Biology, Birkbeck College, University of London, London, United Kingdom; ^3^Division of Biosciences, Institute of Structural and Molecular Biology, University College London, London, United Kingdom

**Keywords:** spider toxin, disulfide bonding, inhibitor cystine knot, penicillamine, peptide misfolding

## Abstract

Peptide toxins that adopt the inhibitory cystine knot (ICK) scaffold have very stable three-dimensional structures as a result of the conformational constraints imposed by the configuration of the three disulfide bonds that are the hallmark of this fold. Understanding the oxidative folding pathways of these complex peptides, many of which are important therapeutic leads, is important in order to devise reliable synthetic routes to correctly folded, biologically active peptides. Previous research on the ICK peptide ProTx-II has shown that in the absence of an equilibrating redox buffer, misfolded intermediates form that prevent the formation of the native disulfide bond configuration. In this paper, we used tandem mass spectrometry to examine these misfolded peptides, and identified two non-native singly bridged peptides, one with a Cys(III)-Cys(IV) linkage and one with a Cys(V)-Cys(VI) linkage. Based on these results, we propose that the *C*-terminus of ProTx-II has an important role in initiating the folding of this peptide. To test this hypothesis, we have also studied the folding pathways of analogs of ProTx-II containing the disulfide-bond directing group penicillamine (Pen) under the same conditions. We find that placing Pen residues at the *C*-terminus of the ProTx-II analogs directs the folding pathway away from the singly bridged misfolded intermediates that represent a kinetic trap for the native sequence, and allows a fully oxidized final product to be formed with three disulfide bridges. However, multiple two-disulfide peptides were also produced, indicating that further study is required to fully control the folding pathways of this modified scaffold.

## Introduction

Disulfide bonds between Cys residues play a key role in stabilizing the conformational properties of peptides and proteins. However, many aspects of the pathways of oxidative protein folding are still unknown, and the relationship between conformational folding and disulfide bond formation remains unclear. Two models for these pathways have been proposed: a “BPTI-like” pathway where all of the folding intermediates contain only the disulfide bonds found in the native structure; and the “hirudin-like” pathway, in which a large number of heterogeneous folding intermediates can form, dominated by intermediates containing non-native disulfide bonds that are rearranged to give the native connectivity (Arolas et al., 2006; Chang, 2011). For many protein architectures, it has been demonstrated that conformational folding drives disulfide bond formation (Welker et al., 2001; Kosuri et al., 2012; Qin et al., 2015; Lv et al., 2018), whereas other proteins adopt intermediate pathways between these two extremes, or can follow different pathways under different conditions (Chang, 2004; Esperante et al., 2017).

**Figure 1:**
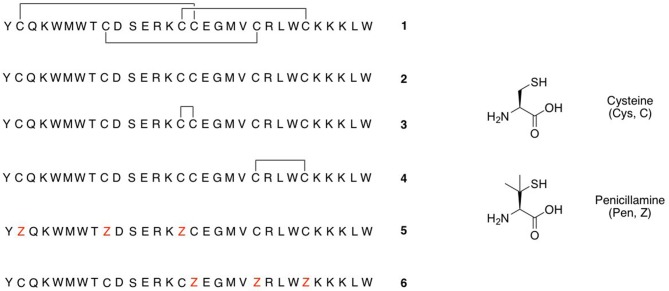
Peptides studied in this paper.

Several families of peptide toxins with extensive networks of cystine bridges have been isolated and characterized from natural sources (Lavergne et al., 2015). These highly constrained peptides with multiple disulfide bridges adopt very stable three-dimensional structures, and as a result have highly specific and potent interactions with biological targets such as ion channels and GPCRs (Ferrat and Darbon, 2005; Akondi et al., 2014; Cardoso and Lewis, 2019). They are thus of significant interest to both pharmaceutical and academic groups, as potential therapeutic leads and as pharmacological probes (Norton, 2017), and one such toxin-derived structure, ziconotide (Prialt) is currently approved for the treatment of severe chronic pain (Schmidtko et al., 2010). One of the most common structural motifs for these toxins is the inhibitor cystine knot (ICK) scaffold, also referred to as a “knottin” fold. These peptides contain six Cys residues connected together by disulfide bonds in a Cys(I)-Cys(IV), Cys(II)-Cys(V), Cys(III)-Cys(VI) connectivity. This arrangement gives rise to an antiparallel beta-sheet structure stabilized by two disulfide bonds, and penetrated by a third disulfide bond that threads through the loop formed by the disulfide bonds and the peptide backbone. Many venom peptides isolated from marine snails of the genus *Conus* (Akondi et al., 2014) and a range of spider and scorpion venom peptides are based on this scaffold, often with the Cys residues in a C-C-CC-C-C pattern (Daly and Craik, 2011; Reinwarth et al., 2012). ICK peptides that show high potency and selectivity as antagonists of the Na_v_1.7 ion channel have recently assumed great importance, as this receptor is a validated target for the treatment of chronic nocioceptive pain (King and Vetter, 2014). Analogs of ProTx-II (1, Figure 1) (Park et al., 2014; Henriques et al., 2016; Flinspach et al., 2017), GpTx-1 (Murray et al., 2015; Chen et al., 2018; Lawrence et al., 2019), JzTx-V (Moyer et al., 2018; Wu et al., 2018), and HwTx-IV (Revell et al., 2013; Agwa et al., 2017) have been identified as potent and selective antagonists of the Na_v_1.7 receptor.

In order to further to probe their structure-activity relationships and to optimize receptor selectivity and potency, many research groups are continuing to investigate analogs of these peptides, either suggested through rational design, or by library screening approaches. Regardless of whether single analogs or libraries are generated by chemical synthesis or recombinant production, the correct folding of the ICK motif, and the right connectivity of the three disulfide bonds, is mandatory for biological activity. However, ensuring that the oxidative folding of linear precursor peptides gives the bioactive isomer is extremely challenging. Orthogonal protecting group strategies, in which each disulfide bridge is formed sequentially from pairs of Cys residues bearing compatible thiol protecting groups (Góngora-Benítez et al., 2014; Heimer et al., 2018a), are time-consuming to achieve and require significant optimization and purification after each step. Whilst aerial oxidation is frequently used when preparing libraries of ICK peptides, for many linear peptide sequences the process is slow and does not give the correct disulfide connectivities (Reinwarth et al., 2013; Wright et al., 2017). Likewise, whilst oxidation with solutions containing DMSO can help to prevent aggregation of the precursor peptides, these can again lead to mixtures of disulfide isomers (Steiner and Bulaj, 2011). Redox buffers containing reduced and oxidized glutathione (GSH/GSSG) or cystine/cysteine are frequently used to promote oxidative folding, as they are believed to reflect *in vivo* oxidation pathways, and can allow equilibration between incorrectly and correctly folded and oxidized intermediates. However, there is no single set of oxidative folding conditions that will work for all peptide sequences, and extensive optimization is frequently required (Steiner and Bulaj, 2011; Reinwarth et al., 2012, 2013; Upert et al., 2014). Understanding the details of the oxidative folding pathways is therefore important for the synthesis of ICK peptides. If the folding pathway that leads to the desired connectivity can be identified, it may then be possible to devise strategies to steer disulfide bond formation toward this pathway and away from pathways that result in misfolding and loss or product. This will ensure high yields and purity of correctly folded ICK peptides.

Studying the folding pathways also presents several challenges. It is usually possible to detect partially folded and oxidized intermediates by HPLC, however on its own this technique cannot identify which cystine bridges have been formed and whether the intermediate species has adopted the native conformation. 2D NMR spectroscopy can elucidate the conformation of some folded intermediates (Le-Nguyen et al., 1993, Heimer et al., 2018b) but frequently the partially oxidized intermediates adopt multiple conformations in solution, and thus the spectral resolution is too poor to allow a structure to be calculated (Čemažar et al., 2003). Mass spectrometry can be used to determine whether one, two or all three disulfide bonds have formed (Wright et al., 2017; Heimer et al., 2018b). In some cases MS/MS sequencing, preceded by sequential partial reduction and alkylation steps, or by enzymatic digestion, can also be used to identify the disulfide connectivities of the intermediate, although this is frequently complicated by over- or under-fragmentation of these complex peptides (Middleton et al., 2002; Heimer et al., 2018a,b). Chemical synthesis of all possible disulfide isomers, using orthogonal protecting group strategies, can also afford insights into the folded structures and ease of formation of non-bioactive isomers (Heimer et al., 2018b), as can MD simulations (George et al., 2018).

So far, few studies have been carried out on linear ICK peptides which fold to give three disulfide bridges, and both BPTI-like and hirudin-like folding mechanisms have been proposed for ICK peptides. Studies of the squash trypsin inhibitor peptide EETI-II folding pathway found a key stable intermediate with native bridges between Cys(II)-Cys(V) and Cys(III)-Cys(VI), with overall the same fold as the bioactive peptide; this is consistent with the BPTI-like model (Le-Nguyen et al., 1993; Reinwarth et al., 2012). It is proposed that this is the direct precursor of the naturally occurring peptide, although it is vulnerable for rearrangement and subsequent mis-folding. It was also demonstrated that replacement of the cysteine residues with serine produced a peptide with native-like secondary structure elements, but without the overall tertiary structure of the native peptide (Heitz et al., 1995). However, a folding study of the Amaranthus α-amylase inhibitor (AAI) peptide observed the formation of a large number of three-disulfide isomers containing non-native disulfide bonds (Čemažar et al., 2003), which is more consistent with a hirudin-like folding pathway. This study also determined the identity of the most abundant folding intermediate (termed MFI), and demonstrated that all three of its disulfide bonds are non-native, including a vicinal disulfide bridge between Cys(III)-Cys(IV), and disulfide bonds between Cys(I)-Cys(II) and Cys(V)-Cys(VI). None of the one- or two-disulfide bonding intermediates could be identified; this was attributed to either their low abundance or their rapid conversion to three-disulfide species (Čemažar et al., 2004).

A promising strategy to induce the correct intramolecular disulfide bonds to form would be to substitute one or more Cys residues with analogs which are structurally similar to Cys but display increased reactivity, either toward another such analog, or toward unmodified Cys residues. Several groups have explored substitution of pairs of Cys residues with pairs of selenocysteine (Sec) residues, which have a higher propensity to form Sec-Sec bonds than Sec-Cys, and have demonstrated that these can be used to form correctly bridged analogs of several ICK peptides, whilst still retaining biological activity (Gowd et al., 2010; de Araujo et al., 2011; Walewska et al., 2011; Steiner et al., 2012). Recently, Wu et al. have established an orthogonal disulfide pairing approach for directed oxidative folding, using the amino acid penicillamine. When peptides which contain both Cys and penicillamine (Pen) residues are cyclized under kinetic oxidative conditions, the formation of Pen-Pen disulfide pairings is disfavoured compared to the more rapidly-formed Cys-Cys or Cys-Pen bonds. Under conditions that allow for disulfide shuffling, at equilibrium Cys-Pen disulfide pairs will be formed in preference to Cys-Cys or Pen-Pen disulfide pairs (Zheng et al., 2015, 2018). Mixed Cys/Pen sequences have thus been used to direct the folding of peptides with three disulfide bridges, and depending on the placement of the Pen residues, oxidative folding can be directed to give mainly the ICK Cys(I)-Cys(IV), Cys(II)-Cys(V), Cys(III)-Cys(VI) disulfide bridging pattern (Zheng et al., 2018).

As part of a study of the substitution of the Cys-Cys bridges in the tarantula venom ICK peptide ProTx-II (1, Figure 1) by the non-reducible mimic lanthionine, we have previously investigated the effects of different conditions for oxidative folding of the linear precursor peptide 2 on the biological activity of ProTx-II (Wright et al., 2017). Using HPLC, nanoelectrospray ionization (nanoESI) mass spectrometry, and ion mobility mass spectrometry (IM-MS) we demonstrated that aerial oxidation of the linear precursor in water gave partial conversion to a mixture of incompletely oxidized isomers. Under these conditions the native isomer with the required Cys(I)-Cys(IV), Cys(II)-Cys(V), Cys(III)-Cys(VI) bridging pattern was not formed, and indeed no fully oxidized isomers with any combination of three disulfide bridges was detected. However, the use of a GSH/GSSG redox buffer was successful in giving predominantly the correct isomer 1 with the biologically active native disulfide bridging pattern (Middleton et al., 2002; Wright et al., 2017).

Whilst folding the peptide in H_2_O did not result in the correctly folded peptide, the misfolded intermediates that were trapped nonetheless carry information about the initial folding pathway of the peptide. In the light of the potential therapeutic importance of ProTx-II, the continuing importance of producing correctly folded libraries of related peptides, and the need to reliably produce analogs in high yield and purity, our motivation for the current study was to use mass spectrometry approaches to investigate the possible folding pathways for linear ProTx-II. In particular, we aimed to identify these incorrectly folded/bridged isomers that arise in the initial folding steps, which could act as possible “kinetic traps.” We also aimed to determine whether incorporating Pen residues at strategic positions could help to direct the folding of the linear precursor to the native disulfide bridging pattern.

## Materials and Methods

### Peptide Synthesis

#### General Methods

Peptide 2, 5, and 6 were prepared by automated solid phase peptide synthesis using a Biotage Initiator+ Alstra™ peptide synthesizer (Biotage AB, Uppsala, Sweden). All peptides were synthesized with an Fmoc protecting group strategy. *N*-α-Fmoc-protected amino acids were purchased from Novabiochem, and used without further purification. Protected amino acids were stored at −20°C. Amino acids carried the following sidechain protecting groups: cysteine, glutamine, penicillamine (Trt); aspartic acid, glutamic acid, serine, threonine, tyrosine (^t^Bu); lysine, tryptophan (Boc); arginine (Pbf). Peptides were synthesized using H-Trp(Boc)-HMPB NovaPEG resin (Novabiochem), and stored at −20°C. Resins were dried overnight *in vacuo* prior to use. HPLC grade *N,N*-dimethylformamide (DMF) (Fischer Scientific) was used as the solvent in all reaction steps. Reagent-grade *N*-methylpyrrolidinone (NMP) (Acros Organics) was used for needle washes. 2-(1H-benzotriazole-1-yl)-1,1,3,3-tetramethylaminium hexafluorophosphate (HBTU) was purchased from Novabiochem. 2,4,6-Collidine was purchased from Thermo Fischer. Reagent-grade piperidine was obtained from Acros Organics. Reagent-grade trifluoroacetic acid (TFA) and ethanedithiol (EDT) were obtained from Sigma Aldrich. Triisopropylsilane (TIPS) was purchased from Alfa Aesar. All reagent quantities were calculated based on the quantity and loading of the resin.

Amino acid solution concentrations were fixed at 0.2 M in DMF, the HBTU solution concentration was fixed at 0.5 M in DMF, and the 2,4,6-collidine solution was fixed at 2 M in DMF. Reagents were dispensed with a syringe needle attached to a robotic arm, which was washed with NMP between reaction steps. Reagents were evacuated from the reaction syringe by application of vacuum for 45 sec. All reactions were carried out at room temperature.

#### Fmoc Deprotection and Coupling Reactions

Fmoc deprotection was achieved by addition of 20% piperidine in DMF (volume equal to half the reaction syringe volume) for 5 min, followed by a fresh addition of an equivalent volume of 20% piperidine in DMF for 10 min. The reactions were mixed at intervals of 10 s on, 15 s off. Following the deprotection, the resin was washed (×6) with DMF.

Amino acid couplings were achieved by addition to the resin of amino acid solution in DMF (5 eq. relative to resin loading), followed by HBTU solution (5 eq.) and 2,4,6-collidine solution in DMF (10 eq.). The coupling reaction proceeded at room temperature over 40 min. The reaction was mixed at intervals of 30 s on, 1 min off. After removal of the coupling mixture, the resin was washed (×2) with DMF, and the coupling step repeated with fresh reagents. After both couplings steps were complete, the resin was washed (×4) with DMF.

#### Peptide Cleavage, Work-Up, and Purification

Prior to cleavage, the peptide resin was washed with DMF (×6), CH_2_Cl_2_ (×6), methanol (×4), and diethyl ether (×4). The resin-bound peptide was dried overnight under vacuum.

Peptides were cleaved from the resin simultaneously with sidechain deprotection using a cleavage cocktail of TFA (94%), TIPS (1%), H_2_O (2.5%), and EDT (2.5%). The cleavage solution was added directly to the dried resin, and shaken at room temperature for 30 min. The resin was filtered and the cleavage solution added directly to a large (>10-fold) excess volume of diethyl ether at −20°C to precipitate peptides. Fresh cleavage solution was added to the resin, and the reaction was shaken at room temperature for 45 min before filtering of the resin and precipitation of peptides, as before.

Precipitated peptides were isolated by centrifugation (15 min, 4,000 g, 4°C) and washed (×3) with a large volume of cold diethyl ether. The pellet was dried in air to remove residual diethyl ether then dissolved in acetonitrile/water and lyophilized.

Semi-preparative HPLC was performed on a Dionex instrument with a PDA-100 photodiode array detector and an ASI-100 automated sample injector, using a ZORBAX 300SB-C18 (5 μm, 9.4 × 250 mm) column (Agilent) with detection at 214 nm and 280 nm. Peptides were separated using a gradient of Buffer B in Buffer A (20–45%) over 30 min at 3 mL/min. Fractions containing the same peptides were pooled and lyophilized.

The quantities of amino acids used for the synthesis of each peptide, yields, and analytical HPLC traces of the pure peptides, are given in the Supplementary Material.

### Oxidative Peptide Folding

Folding of each peptide was conducted using two methods:

#### Folding in GSSG/GSH Buffer

Linear peptide 2 was dissolved to 0.1 mg/mL in a buffer solution containing 2 M urea, 0.1 M Tris-HCl, 0.15 mM reduced glutathione, and 0.3 mM oxidized glutathione. The solution was adjusted to pH 8 using the gradual addition of NaOH and then left shaking at room temperature for 24 hr. On completion, the solution was adjusted to pH 3 with HCl before analysis by HPLC.

#### Folding in H_2_O

Linear peptide 2 was dissolved to 0.1 mg/mL in HPLC-grade water, and allowed to oxidize over 7 days at 4°C. The peptide solution was then lyophilized.

#### Analytical HPLC Methods

Analytical HPLC was performed on an Agilent 1260 Infinity instrument with a Reprosil Gold 200 C8 (5 μm, 4.6 × 250 mm) column (Dr. Maisch GmbH) and equipped with a 10 mm guard column. Analysis was performed using a 5–75% gradient over 60 min of Buffer B (acetonitrile + 0.1% TFA) in Buffer A (water + 0.1% TFA) at a flow rate of 1 mL/min with detection at 214 nm.

#### Alkylation of Cysteine With *N*-Ethylmaleimide

A buffer solution containing *N*-ethylmaleimide (NEM) (40 mM) and ammonium formate (50 mM) was made and adjusted to pH 3.5 with formic acid. The remaining peptide samples were dissolved in water (50 μL) before addition of the NEM buffer (50 μL) and allowed to react for 20 min. The solution was then diluted with 0.1% TFA in water (200 μL) and desalted on pre-equilibrated Sep-Pak C18 cartridges by washing with 5% MeCN/ 0.1% TFA (2 × 400 μL) and eluting with 80% MeCN/0.1% TFA (1 × 200 μL). The eluted peptide solution was lyophilized. Disulfide bonds were reduced by reaction with TCEP (10 μL, final concentration 5 mM) over 3 hr.

### Mass Spectrometry

Low-resolution mass spectrometry was performed using a Waters Acquity UPLC SQD LC-MS instrument equipped with a Hypersil GOLD C4, 5 μm particle, 50 × 2.1 mm column (Thermo Scientific). Water (+0.1% formic acid) and acetonitrile (+0.1% formic acid) were used as solvents with a linear gradient of 5–95% acetonitrile across 9 min with a flow rate of 0.3 mL/min. Spectra were acquired in positive electrospray ionization mode across a 100–2,000 m/z range, and data were analyzed using MassLynx.

High-resolution mass spectrometry was carried out using an Agilent 6510 QToF LC-MS instrument. Samples were injected onto an Agilent 300Extend-C18 column (150 × 2.1 mm, 3.5 μm particle). Water (+0.1% formic acid) and acetonitrile (+0.1% formic acid) were used as solvents with a gradient elution of 5–95% acetonitrile over 10 min and flow rate of 0.3 mL/min. The source parameters for gas temperature, gas flow and nebulizer were set to 325°C, 51/min and 20 psig, respectively. The column effluent was passed into the capillary ESI source of the mass spectrometer and mass spectra were acquired in positive ion mode using the m/z range 1,000–3,000 in profile mode. Data were analyzed using MassHunter.

### Peptide Identification

Peptides were analyzed on a ThermoFischer Q Exactive Hybrid Quadrupole-Orbitrap mass spectrometer connected to a Vanquish pump and autosampler (Thermo Fischer). Peptides were separated using a TR-5MS (30 × 0.25 mm) column using a gradient of 5–55% acetonitrile (0.1% formic acid) in water (0.1% formic acid) over 90 min.

MS1 scans (200–2,000 m/z) were set to a resolution of 70,000 (at 200 m/z) and AGC target of 3e6. The maximum IT was set to 100 ms. MS2 scans were triggered by an ion intensity exceeding 1e3, and the top 5 peaks meeting this criterion were isolated (isolation width 4 m/z). Ions with charge states <4 were not considered for MS2. Precursor ions appeared in the MS1 spectrum with charge states between +4 and +7 and MS2 spectra for all the different charge states were collected to ensure complete detection of fragments. Peptide matching was set to preferred and isotope exclusion was set to on. Dynamic exclusion was turned off. Peptides were fragmented in HCD mode with a stepped NCE of 25-30-35. MS2 scans (200–4,000 m/z) were set to a resolution of 17,500 and AGC target of 1e5. The maximum IT was 50 ms. Data were analyzed using XCalibur (Thermo Fischer), and peptide fragment ions were assigned manually. Fragments were considered a match if the charge state was determined to be correct and the mass accuracy was <10 ppm.

## Results and Discussion

### Analysis of Oxidative Folding Pathways of the Native ProTx-II Sequence

We have previously observed that the oxidative folding of ProTx-II using aerial oxidation in the absence of a redox buffer did not produce fully folded peptide (Wright et al., 2017). We hypothesize that this is likely to be due to the formation of misfolded, incorrectly bridged disulfide isomers which are trapped under the kinetic conditions of the reaction. While this therefore makes folding in water inappropriate as a synthetic route to this particular ICK peptide, the trapped species produced in the experiment can nonetheless provide useful information about the initial folding paths of the peptide. Identifying these misfolded peptides would allow a better understanding of the complex ICK peptide folding pathway, and perhaps hint at improved methods to fold these peptides.

**Figure 2:**
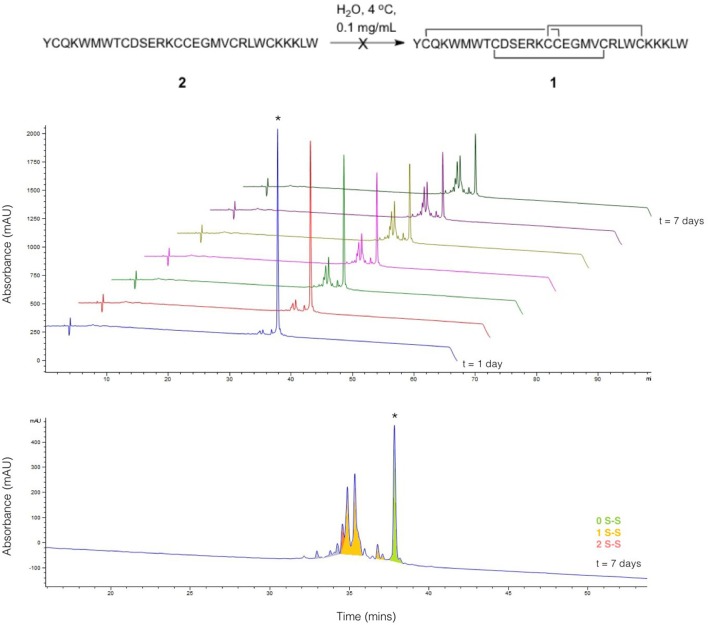
Folding of linear ProTx-II under aerial oxidation conditions in water. (Top) overlay of analytical HPLC chromatograms showing folding of linear ProTx-II over 7 days; the linear peptide is indicated by *. (Bottom) analytical HPLC for the products of folding after 7 days; peaks are color-coded by the number of disulfide bonds in the product, as determined by mass spectrometry. The small peaks in purple correspond to species with oxidized methionine.

Linear ProTx-II 2 was synthesized and purified as previously described (Wright et al., 2017), and allowed to oxidize in H_2_O at 4°C over the course of a 7-days period. Aliquots were collected each day and analyzed by HPLC (Figure 2). The appearance of folded peptides with lower retention times can clearly be seen, together with a corresponding decrease in intensity of the linear peptide peak. The product peaks show a steady increase over the experiment timescale: no peaks are seen which show an increase in intensity, followed by a decrease. This implies that all the peaks seen in the chromatogram correspond to stable, folded peptides, and are not intermediates to other products. These can therefore be treated as trapped misfolded peptides that are unable to oxidize further. The mixture of peptides produced after 7 days of folding was analyzed by mass spectrometry to identify the number of disulfide bonds in each peak. As was previously observed (Wright et al., 2017), no peptides with three disulfide bonds were found. Only one product peak had two disulfide bonds; the remaining peaks all had one disulfide bond.

**Figure 3:**
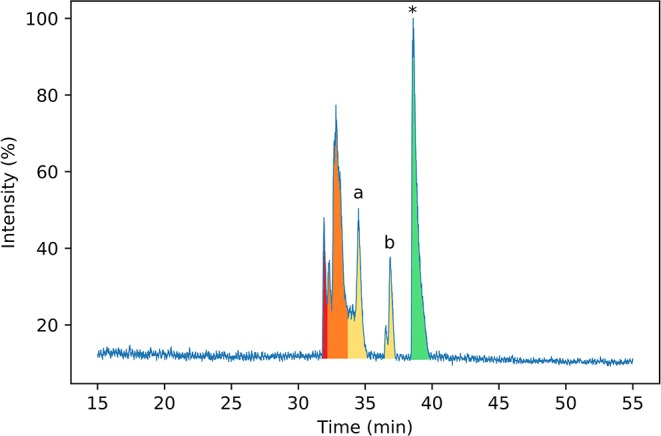
Normalized base peak intensity (BPI) chromatogram determined by LC-MS/MS for the products of folding linear ProTx-II 2 in water after 7 days. Peaks are color-coded by the number of disulfide bonds in the peptide (as determined by MS1 scans). Peptides with two disulfide bonds are colored in red; peptides with one disulfide bond are colored in yellow, and peptides with zero disulfide bonds are colored in green. Peaks containing mixed one- and two-disulfide peptides are colored orange. Peptide 3 corresponds to peak (a), while peptide 4 corresponds to peak (b). Linear ProTx-II is indicated by *.

To identify the disulfide bond pairings in these species, the peptide mixture was analyzed by LC-MS/MS. A long chromatography gradient was required to separate the peptides, which were then sprayed directly into the mass spectrometer. The chromatogram for the peptide mixture analyzed using LC-MS/MS (Figure 3) had a similar profile to that from the analytical HPLC shown in Figure 2.

**Figure 4:**
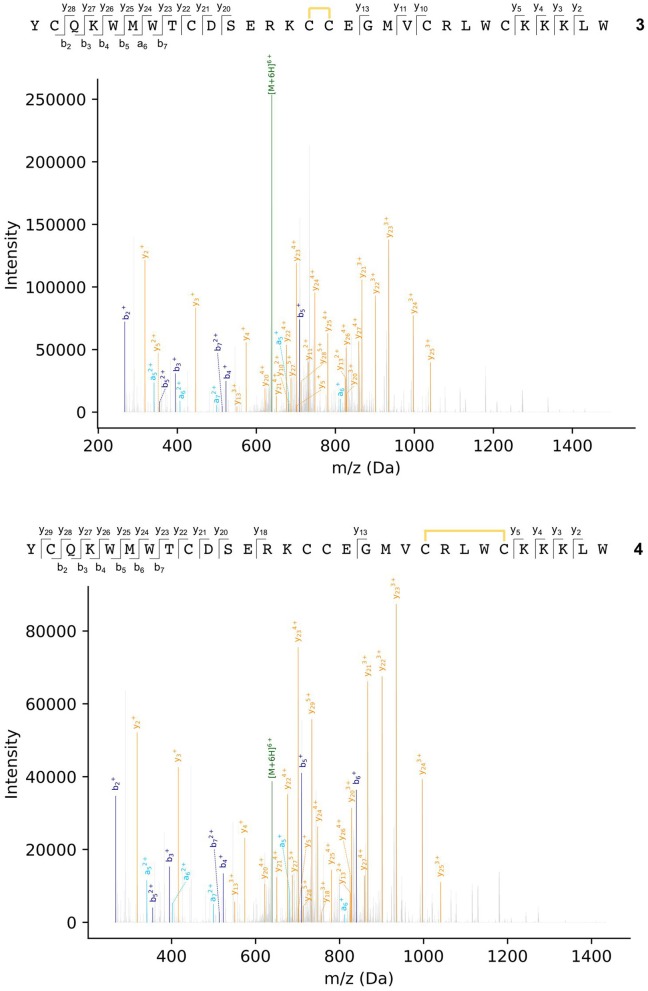
MS2 spectra of ProTx-II folding intermediates 3 (top) and 4 (bottom). Disulfide bonds are indicated by yellow lines. Details of fragment assignments are provided in the Supplementary Material.

Peptides were subjected to higher-energy collisional dissociation (HCD) fragmentation followed by detection in the Orbitrap mass analyser. A stepped collision energy was used to maximize fragment coverage (Diedrich et al., 2013). Two peptides, each with one disulfide bond, could be identified from the MS2 spectra from peaks a and b in the chromatogram (Figure 4), where fragments containing a disulfide bond could be identified by a mass shift (−2.01565 Da) compared to the linear peptide. One peptide (3) contains a vicinal disulfide bond [Cys(III)-Cys(IV)] while the other (4) contains a disulfide bond between the two C-terminal cysteines [Cys(V)-Cys(VI)]. Incomplete fragmentation (especially in the b-ion series) and overlapping chromatographic peaks made complete identification of the disulfide bonding in some of the peptides impossible. However, in all cases the strong y-ion series, and presence of the b_2_ ion, near the *N*-terminus demonstrated that Cys(I) and Cys(II) were not involved in disulfide bonding.

In order to produce more complete fragmentation data, and extend the number of peptides that could be identified, a disulfide mapping experiment was attempted (Albert et al., 2016). The partially bridged peptides were isolated by HPLC, and then subjected to alkylation using the cysteine-specific alkylating reagent *N*-ethylmaleimide (NEM), followed by reduction by TCEP and analysis by mass spectrometry. However, the peptide fragmentation [especially between the vicinal Cys(III) and Cys(IV)] was still insufficient to confidently identify the disulfide bonding in the remaining peptides.

Both of these mass spectrometry experiments suggest that the initial folding of linear ProTx-II 2 occurs exclusively at the C-terminus of the peptide as in the singly bridged intermediates 3 and 4, Cys(I) and Cys(II) are not disulfide bonded. This is consistent with the findings of Le-Nguyen et al. (1993) who suggested that the *N*-terminal Cys(I) is the last to form its native disulfide bond. This in turn suggests two possible pathways for kinetic folding of this ICK peptide: one in which a vicinal disulfide bond initially forms between [Cys(III)-Cys(IV)] and one where the first bond forms between [Cys(V)-Cys(VI)]. Vicinal disulfide bonds are unusual among stably-folded proteins as they impose tight conformational restrictions on the peptide backbone immediately surrounding the disulfide (Richardson et al., 2017). However, they have been described before in previous naturally-occurring peptides (Wang et al., 2000; de Araujo et al., 2013). Two consecutive cysteines benefit from a high local concentration which makes the formation of a disulfide between them much more likely. Additionally, the peptide backbone is unstructured in unfolded peptides, which makes the conformational requirements of a vicinal disulfide much less onerous. Once formed, strained disulfide bonds such as vicinal disulfide bonds tend to be more reactive to disulfide shuffling or reduction (Karimi et al., 2016). Indeed, when oxidative folding of the ICK peptide AAI was carried out in a redox buffer, the non-natural [Cys(III)-Cys(IV)] vicinal disulfide bridge was observed in several folding intermediates, including the most abundant intermediate, and presumed to rearrange under equilibrating conditions to the bioactive peptide (Čemažar et al., 2003). However, under kinetic (irreversible conditions) such as the aerial oxidation conditions used for ProTx-II, shuffling and/or reduction of the initially formed [Cys(III)-Cys(IV)] intermediate is suppressed. It is likely that the conformational restriction imposed on the peptide by the vicinal disulfide bridge impedes the formation of further disulfide bonds.

Turning to the other singly bridged intermediate, the presence of a peptide containing the disulfide bond at the C-terminus between Cys(V) and Cys(VI) may indicate an important role for the C-terminus in the folding of ProTx-II. It may imply that the initial folding of the peptide is occurring exclusively at the C-terminus, thus bringing the Cys(V) and Cys(VI) residues into close proximity during folding. This is plausible as the Cys(V)/Cys(VI) C_α_-C_α_ distance in the X-ray crystal structure of the fully-folded peptide is just 4.613 Å (Wright et al., 2017). Both Cys(V) and Cys(VI) are found in the β-hairpin region of ProTx-II. Since the β-hairpin appears to be a highly-conserved feature of ICK peptides and secondary structure elements are expected to form more quickly than tertiary folds (Srinivasan and Rose, 1999) it can be suggested that the formation of this turn is a key initial step in the folding of ProTx-II, and acts as an initiation site for oxidative folding. This has been suggested to be a key step in the folding of EETI-II (Le-Nguyen et al., 1993; Wentzel et al., 1999).

**Scheme 1:**
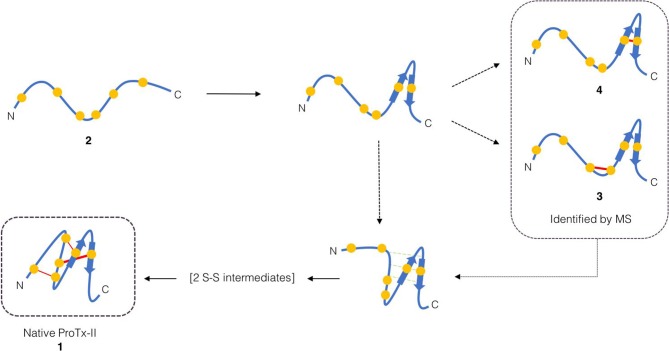
Proposed initial folding pathways for ProTx-II. Cysteine residues are represented by yellow circles, and disulfide bonds by red lines. The unfolded linear peptide 2 first forms a β-hairpin, which brings Cys(V) and Cys(VI) into proximity and permits formation of trapped peptide 4. The vicinal cysteine residues Cys(III) and Cys(IV) are able to form disulfide-bonded peptide 3 independently of the β-hairpin formation. Formation of the β-hairpin allows hydrogen bonding interactions (indicated in green) with a second loop of the peptide backbone. We then conjecture that, under redox conditions, this permits disulfide bond formation (by oxidation or shuffling) in the C-terminal cysteines to form two-disulfide intermediates. The *N*-terminal loop can then be brought around to form the Cys(I)-Cys(IV) disulfide bond and complete the folding.

Based on this observation, and analysis of hydrogen bonding in the X-ray crystal structure of ProTx-II, some initial steps in the folding of ProTx-II can be proposed (Scheme 1). In this model, the C-terminal β-hairpin is the first region of the peptide to fold. The Cys(V) and Cys(VI) residues are brought into proximity on the same face of the β-hairpin, and a disulfide-trapped peptide with the [Cys(V)-Cys(VI)] connectivity was identified by mass spectrometry. The vicinal Cys(III) and Cys(IV) (which are unlikely to be affected by the formation of the β-hairpin) are able to form a disulfide bond, which was also identified in the oxidative folding experiment.

**Figure 5:**
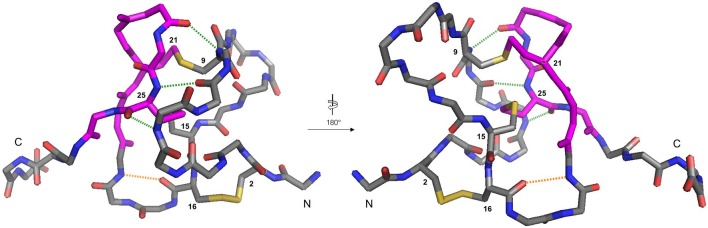
Backbone traces of the X-ray crystal structure of ProTx-II (PDB: 5O0U) showing key hydrogen-bonding interactions. For clarity, sidechains are hidden, except for disulfide bonds, and cysteine residues are numbered. Residues involved in the β-hairpin region are colored magenta. The Cys16-Gly18 hydrogen bond is displayed in orange, and the Met6-Cys25, Trp7-Cys25, and Cys9-Leu23 hydrogen bonds are displayed in green. The N- and C-termini are labeled.

It is likely that the formation of this β-hairpin allows further hydrogen bonds to form between the backbone atoms of Leu23 and Cys25 and residues Met6, Trp7, and Cys9 (Figure 5, green dashed lines); in some ICK peptide structures (such as Guangxitoxin-I and Hainantoxin-I) this region of the peptide forms a third strand in a formal β-sheet (Li et al., 2003; Lee et al., 2010). A flexible Gly18 residue at the base of the β-hairpin, and a hydrogen bond between Cys16 and Gly18 (Figure 5, orange dashed line) assists in the formation of this second loop. In this conformation, Cys(II), Cys(III), Cys(V), and Cys(VI) are well-positioned to form native disulfide bonds, and under redox conditions any non-native disulfide bonds that have formed can be reshuffled into the native conformation. From this conformation, we hypothesize that the *N*-terminus is looped around, supported by the formation of hydrogen bonds between the backbone of Gln3 and Lys14, and also between Lys4 and Trp7. The final disulfide bond [Cys(I)-Cys(IV)] is then able to form, either by oxidation of the cysteine sidechain sulfhydryls, or by disulfide shuffling.

### Analysis of the Oxidative Folding Pathways of Analogs of ProTx-II Containing Pen

Based on these observations, we hypothesized that it might be possible to direct the folding pathway of the ProTx-II sequence to the correct ICK fold and connectivity by adding Pen residues at strategic positions. Under oxidative folding conditions the formation of Pen-Pen disulfide bonds is disfavoured (Zheng et al., 2017), and under equilibrium conditions Pen-Cys disulfide bonds are thermodynamically favored. Replacing three of the Cys residues with Pen should provide the largest possible reduction in disulfide isomers (from 15 to 6 possible isomers). Additionally, as the Pen-Cys disulfide bond has been reported to be significantly more reductively stable than the naturally occurring Cys-Cys disulfide bond, due to steric inhibition of thiol-disulfide exchange, so peptides designed on this basis might also exhibit greater *in vitro* stability (Zheng et al., 2015). Accordingly, we designed modified peptides such that three Pen residues were designed to pair with the Cys residues found in the correct bioactive connectivity. Of the possible isomers satisfying this condition, we elected to synthesize two peptides, [Pen-Pen-Pen-Cys-Cys-Cys] 5 and [Cys-Cys-Cys-Pen-Pen-Pen] 6 (Figure 1). Both peptides were synthesized using standard peptide coupling conditions (Supplementary Material), with Fmoc-Pen(Trt)-OH used to replace Fmoc-Cys(Trt)-OH where required. The synthesis of both peptides proceeded in only moderate yield and purity, owing to the difficulties inherent in incorporation of the more sterically hindered, and conformationally restricted, Pen residues (van Woerkom and van Nispen, 1991; Rajarathnam et al., 1999; Tran et al., 2006). Thus, in addition to the desired peptides 5 and 6, multiple deletion sequences, lacking one, two or three Pen residues were isolated during HPLC purification (Supplementary Material) and identified by mass spectrometry.

**Figure 6:**
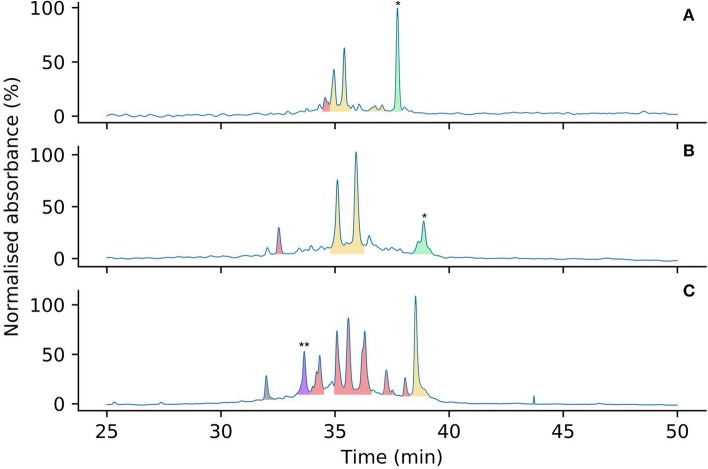
Analytical HPLC chromatograms comparing the oxidation product profiles of 2 **(A)**, 5 **(B)** and 6 **(C)** after 7 days in water, color coded by number of disulfide bonds (determined by mass spectrometry). Linear peptides are indicated by *. Peaks corresponding to peptides containing zero (green), one (yellow), two (red), and three (purple) disulfide bonds are highlighted by color. Mass spectra to support the assignment of the three-disulfide peptide indicated by ** are provided in the Supplementary Material. The peak in gray is an unknown small molecule, possibly a degradation product.

To compare the effects of Pen incorporation on the initial folding pathway of these peptides, and to investigate whether these substitutions prevented the formation of unproductive kinetic traps, both [Pen-Pen-Pen-Cys-Cys-Cys] 5 and [Cys-Cys-Cys-Pen-Pen-Pen] 6 were allowed to oxidize in water under the same conditions as for wild-type Protoxin-II 2 in Section Analysis of Oxidative Folding Pathways of the Native ProTx-II Sequence (0.1 mg/mL peptide at 4°C over 7 days). After 7 days the oxidative folding reaction products were analyzed by HPLC (Figure 6), the peaks isolated and the number of disulfide bonds in each folded species was determined by mass spectrometry. Under these conditions, both wild-type ProTx-II peptide 2 (Figure 6A) and [Pen-Pen-Pen-Cys-Cys-Cys] 5 (Figure 6B) produced peaks containing only partially oxidized structures, with some of the linear peptide also remaining. Both peptides produce a similar product profile in the analytical HPLC chromatograms, suggesting that substitution of Cys by Pen at the *N*-terminus has not drastically affected the folding pathway. Conversely, [Cys-Cys-Cys-Pen-Pen-Pen] 6 had a markedly different profile with multiple well-resolved peaks being observed (Figure 6C). Moreover, one of these peaks corresponds to a fully oxidized isomer in which three disulfide bonds have been formed. This has never previously been achievable with the wild type sequence 2 under aerial, non-equilibrating conditions.

Overall, it is clear that Pen incorporation is not, in itself, completely sufficient to direct folding of the peptide toward the fully folded, three disulfide form, as even in the case of [Cys-Cys-Cys-Pen-Pen-Pen] 6, the majority of the peaks contain just two disulfide bonds. It is however intriguing to find that placing Pen residues at the *N*-terminus or the *C*-terminus of the peptide produces significantly different results. If the initial folding of wild-type ProTx II 2 occurs primarily at the C-terminus of the peptide, as suggested by Figures 3, 4 and previous studies of related disulfide-rich peptides (Le-Nguyen et al., 1993) then placing Pen residues in the Cys(I) and Cys(II) positions, as in [Pen-Pen-Pen-Cys-Cys-Cys] 5, will not improve the selective folding.

Of the two possible pathways that we have suggested for kinetic folding of ProTx-II type peptides (Scheme 1), the pathway where the first bond forms between Cys(V)-Cys(VI) would be effectively prevented in [Cys-Cys-Cys-Pen-Pen-Pen] 6 due to the unfavorable sterics of a Pen(V)-Pen(VI) disulfide bond. This appears to allow the partially folded peptide to avoid this particular kinetic trap and progress past this point to the formation of a fully oxidized final product. However, it is possible that once the peptide is able to progress past this one-disulfide kinetic trap, new two-disulfide traps are forming, which can be seen in the folding profile (Figure 6).

In addition, it is unclear whether the pathway where the first bond forms between the vicinal Cys(III)-Cys(IV) residues (Scheme 1) would be prevented in [Cys-Cys-Cys-Pen-Pen-Pen] 5. Cyclic Cys-Pen dipeptides have been previously reported (Baxter et al., 1988; Cumberbatch et al., 1993) and shown to contain cis-amide bonds. However, macrocyclisation to form such medium sized eight-membered rings is entropically disfavoured, the resulting rings are strained (Ruggles et al., 2009), and it is not known whether vicinal cyclic Cys-Pen structures in larger peptides are more, or less, favored than vicinal cyclic Cys-Cys structures, as this motif has not previously been investigated.

To investigate the effect of redox oxidation conditions on the folding of 5 and 6, each peptide was stirred in a buffer containing GSH/GSSG for 24 h, following the previously published procedures (Park et al., 2014; Wright et al., 2017). Unlike the linear wild-type ProTx-II 2, both peptides were seen to partially precipitate out of solution under these conditions. However, despite the loss of a large portion of the Pen-containing peptides under the buffer conditions, enough remained in solution for fully-oxidized peptides [Pen-Pen-Pen-Cys-Cys-Cys] 5 and [Cys-Cys-Cys-Pen-Pen-Pen] 6 to be detected and isolated by HPLC. Mass spectrometry confirmed that these folding products contained three disulfide bonds, consistent with the results found for the wild-type Protoxin-II (Supplementary Material).

## Conclusions

Selection and optimization of the oxidative folding conditions is crucial to the successful synthesis of ICK peptides with the correct disulfide connectivities and bioactive fold. Previous studies have indicated plausible oxidative folding pathways for some related disulfide-rich peptides, and the structures of some possible folding intermediates, and probable kinetic traps, have been determined. However, there is no consensus pathway for the oxidative folding of the ICK family as a whole. Indeed, it is probable that more than one pathway can operate, and that the tendency for a given linear sequence to follow a particular pathway is highly sequence-dependent. Unambiguous identification of productive folding intermediates and possible kinetic traps is crucial to understanding the folding pathways in operation. However, under redox buffer conditions, which are intended to promote rapid disulfide formation and shuffling, experimenters cannot be sure that any observed intermediates (which are rapidly interconverting) truly represent the initial stages of the folding, as it is possible that several rapid disulfide formation and shuffling steps have already taken place on a timescale too rapid to be observed. This is supported by recent studies of ICK folding (Čemažar et al., 2003) in which only intermediates with three disulfide bonds could be identified; presumably these did not form simultaneously, but arose from stepwise oxidation through unidentified one- and two-disulfide intermediates. By contrast, folding under irreversible conditions (aerial oxidation in water) lowers the rate of disulfide bond formation and greatly suppresses disulfide shuffling; thus, the intermediates observed in these experiments represent the very first steps in the folding pathway.

In view of the therapeutic importance of ProTx-II, we have studied the initial stages of misfolding of the linear peptide under aerial oxidation conditions in water. Using HPLC and mass spectrometry, we have identified and characterized for the first time two peptides with just a single, non-native disulfide bond. We have showed that initial disulfide bond formation is focused on the *C*-terminus of the peptide, and, based on these results and existing data on ICK peptide folding, we propose a model for the folding of ProTx-II centered around the formation of the *C*-terminal β-hairpin. The formation of the one-disulfide intermediates containing non-native disulfide bonds is consistent with both the hirudin-like folding pathway identified for AAI; in fact the Cys(III)-Cys(IV) and Cys(V)-Cys(VI) disulfide bonds found in our intermediates 3 and 4 are also present in the MFI intermediate. However, the AAI study did not identify any of the one- or two-disulfide intermediates, leaving the mechanism of the formation of the MFI intermediate an open question. Our results indicate that the formation of such intermediates in ProTx-II is focused around the *C*-terminus, which is consistent with the predominant folding intermediate found for EETI-II. The proposed folding scheme for ProTx-II therefore shows elements of both folding pathways. As the key elements of the hypothesis (the final disulfide connectivity, the presence of the β-hairpin, and the backbone hydrogen-bonding interactions) are conserved across many ICK peptides, it can be suggested that this folding scheme may also apply to other ICK peptides and not just ProTx-II.

This hypothesis suggests ways to direct the folding of ProTx-II away from the Cys(V)-Cys(VI) misfolding trap and toward native disulfide bond connectivity. We have studied whether it is possible to do this by the strategic placement of Pen residues to direct the oxidative folding toward the bioactive connectivity. We have shown that the exact placement of the Pen residues plays a key role in directing the folding of ICK peptides without the use of redox agents. Folding of a ProTx-II variant, modified to contain three Pen residues at the *N*-terminus, results in a folding product profile similar to the wild-type peptide. There are two main isomers, which each contain a single disulfide bond and are hypothesized to be kinetic traps that prevent full folding of the peptide. Conversely, in the variant with three Pen residues at the *C*-terminus only a single one-disulfide isomer is observed suggesting that the Cys(V)-Cys(VI) kinetic trap has been avoided. In fact, with this placement of the three Pen residues, we have prepared for the first time a ProTx-II analog which was able to fold into a structure containing three disulfide bonds under irreversible aerial oxidation conditions. However, multiple two-disulfide peptides were also produced when folding this peptide, suggesting that there is more to be understood when considering how to obtain the fully folded structure with high yield. In addition, although our results suggest that these variants follow a similar folding pathway to ProTx-II under redox conditions, it will be necessary to confirm the disulfide connectivity and three-dimensional structure to fully understand the influence of Pen on the folding.

The introduction of Pen allows the experimenter the ability to influence the disulfide bond connectivity, but also carries the risk of reducing bioactivity of the resulting peptides. Both increases and decreases in receptor binding potency have been observed on substitution of Pen residues in biologically active peptides (Flippen-Anderson et al., 1994; Bélec et al., 2001) binding and selectivity are altered/reduced. For variants of multiply bridged peptides the positioning of the Cys-Pen bridge is critical, with some variants showing reduced or negligible receptor binding despite the correct bridging pattern being achieved (Hunt et al., 1993; Fraszczak et al., 2002), whereas other variant peptides with Cys residues replaced by Pen showed excellent biological activity (Jaskiewicz et al., 1998). Understanding the bioactivity of Pen-variant peptides will thus require knowledge of the effect that the Pen residue(s) have on the overall peptide conformation as well as the steric effects of dimethyl substitution on receptor binding.

Previous work has shown that orthogonal Pen-Cys pairing can promote the desired selectivity to moderately high yields in artificial peptides. However, this study has critically revealed the complexity of using Pen in natural peptides with more complicated oxidative folding pathways. In this case, it is not enough to simply place Pen residues to promote native disulfide connectivity, but one must also actively disfavor disulfide bonds that lead to kinetic traps. Considering that there are many peptide toxins that fall into similar kinetic traps as those discussed here, understanding how to place Pen to avoid these traps could be important both in terms of fundamental studies of peptide folding and also as a route to new lead compounds as selective ion channel blockers.

## Data Availability Statement

All datasets generated for this study are included in the article/Supplementary Material.

## Author Contributions

JM and SM synthesised linear peptide 2. SM was responsible for designing and carrying out the oxidative folding studies on the linear peptide 2. JR was responsible for the synthesis of the penicillamine-containing peptides 5 and 6, and for designing and carrying out the studies on the oxidative folding pathways of these peptides. SM carried out the mass spectrometry experiments, with input from KT. SM, JR, and AT analyzed the results. AT led the writing of the publication. All authors discussed the project and assisted with the writing of the manuscript.

### Conflict of Interest

The authors declare that the research was conducted in the absence of any commercial or financial relationships that could be construed as a potential conflict of interest.
