# Supplementary material for: A Chemical Biology Approach to Probing the Folding Pathways of the Inhibitory Cystine Knot (ICK) Peptide ProTx-II
Source: Front Chem. 2020 Apr 3;8:228. doi: 10.3389/fchem.2020.00228 (PMC7145985; doi:10.3389/fchem.2020.00228)
Supplement: Supplementary file 1 [file Data_Sheet_1.PDF]

Supplementary Material

Contents

S1 Quantities and Characterisation of peptides 2
S2 Fragmentation data for MS/MS experiments 7
S3 Mass spectra for buffer-folded penicillamine peptides 10
S4 Mass spectrometry of 3xS-S product from folding of peptide 6 in water 12
S5 Supplemental tandem mass spectra 13
S6 Alkylation/reduction of coeluting one- and two-disulfide peptides 14

S1 Quantities and Characterisation of peptides

Peptide **2**

Y C Q K W M W T C D S E R K C C E G M V C R L W C K K K L W **2**

Scale: 0.082 mmol
Isolated yield: 15%

Amino acid	Mass (g)	Dissolved volume (mL)
Fmoc-Cys(Trt)-OH	3.471	26.777
Fmoc-Lys(Boc)-OH	2.316	22.834
Fmoc-Arg(Pbf)-OH	1.292	8.900
Fmoc-Trp(Boc)-OH	1.567	13.595
Fmoc-Asp(^{t}Bu)-OH	0.415	4.705
Fmoc-Leu-OH	0.704	9.398
Fmoc-Ser(^{t}Bu)-OH	0.386	4.729
Fmoc-Tyr(^{t}Bu)-OH	0.463	4.663
Fmoc-Glu(^{t}Bu)-OH	0.847	9.274
Fmoc-Met-OH	0.740	9.367
Fmoc-Thr(^{t}Bu)-OH	0.401	4.717
Fmoc-Gly-OH	0.300	4.805
Fmoc-Val-OH	3.420	4.768

Table S1.1*: Amino acid quantities used in the synthesis of peptide* ***2***

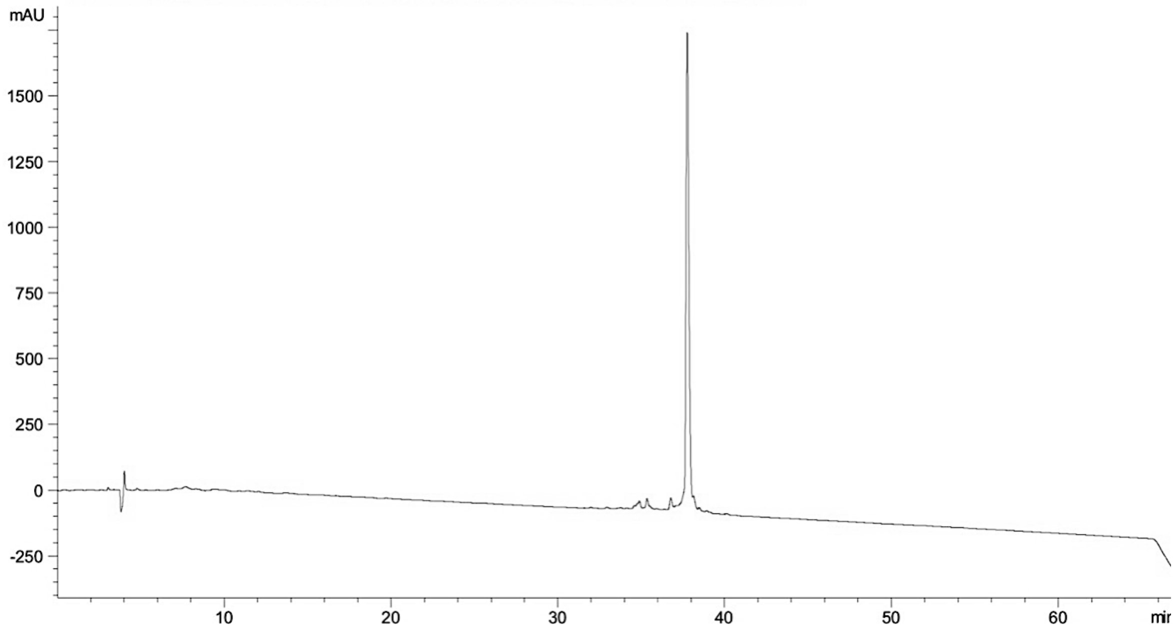

Figure S1.1*: Analytical HPLC for purified peptide* ***2***

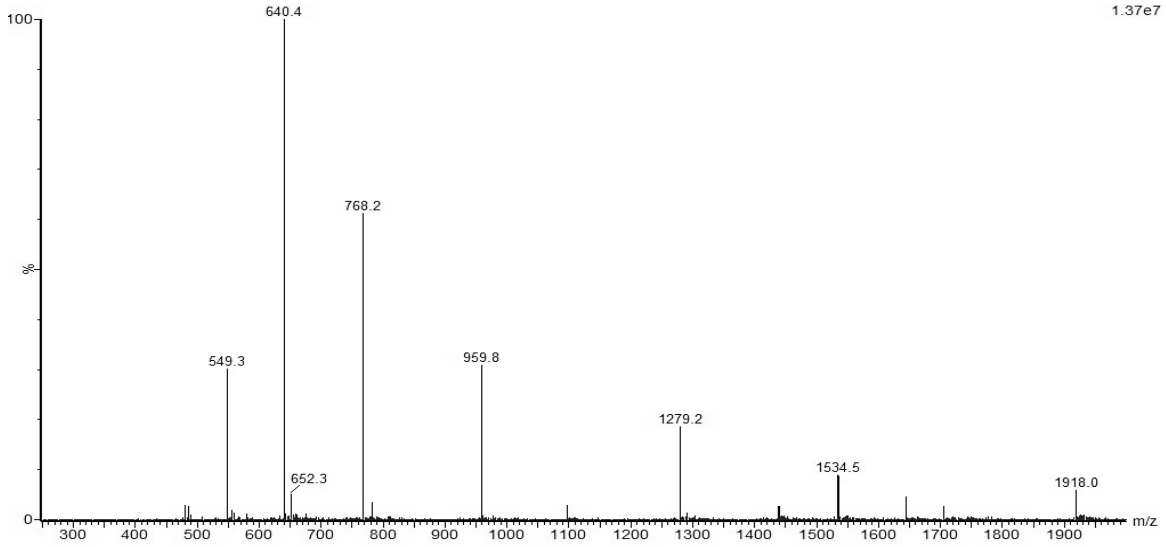

Figure S1.2**: Mass spectrum of purified peptide **2

Peptide **5**

Y Z Q K W M W T Z D S E R K Z C E G M V C R L W C K K K L W **5**

Scale: 0.041 mmol
Isolated yield: 7.2%

Amino acid	Mass (g)	Dissolved volume (mL)
Fmoc-Cys(Trt)-OH	0.878	6.775
Fmoc-Lys(Boc)-OH	1.164	11.472
Fmoc-Arg(Pbf)-OH	0.654	4.499
Fmoc-Trp(Boc)-OH	0.790	6.852
Fmoc-Asp(^{t}Bu)-OH	0.212	2.408
Fmoc-Leu-OH	0.356	4.756
Fmoc-Ser(^{t}Bu)-OH	0.198	2.422
Fmoc-Tyr(^{t}Bu)-OH	0.239	2.387
Fmoc-Glu(^{t}Bu)-OH	0.428	4.693
Fmoc-Met-OH	0.374	4.740
Fmoc-Thr(^{t}Bu)-OH	0.205	2.414
Fmoc-Gly-OH	0.154	2.400
Fmoc-Val-OH	0.175	2.441
Fmoc-Pen(Trt)-OH	0.920	6.739

Table S1.2**: Amino acid quantities used in the synthesis of peptide **5

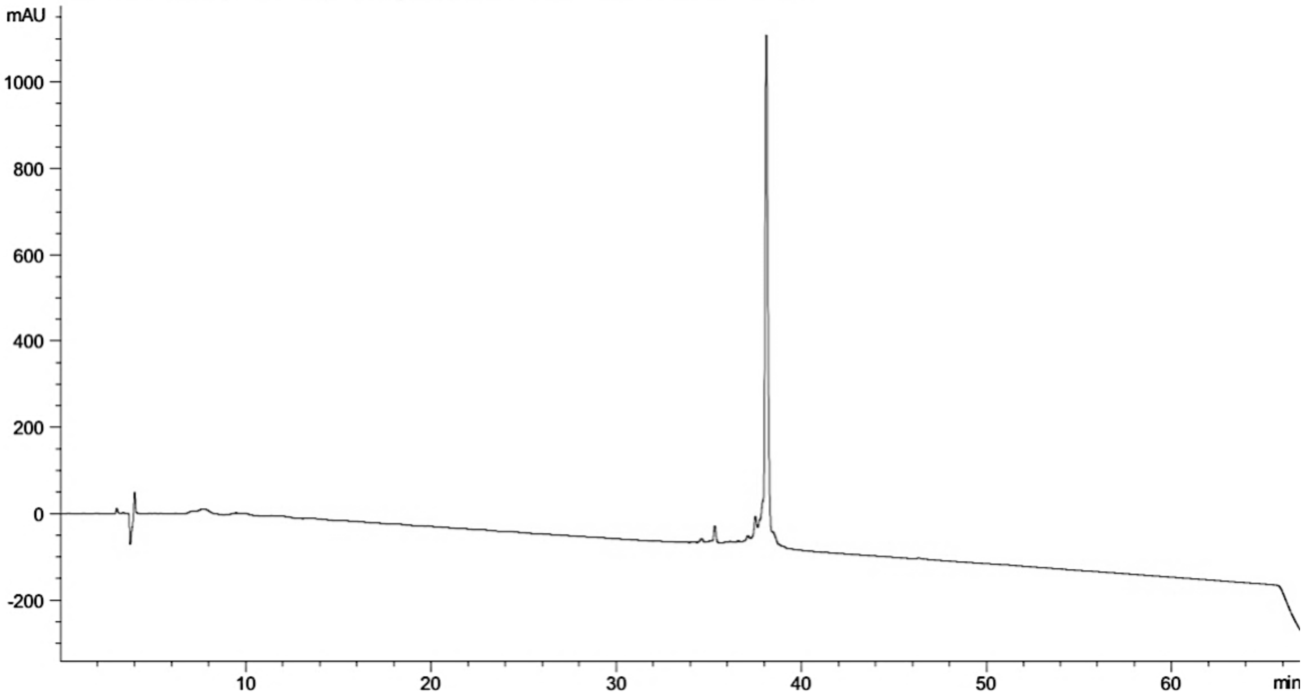

Figure S1.3**: Analytical HPLC trace for purified peptide **5

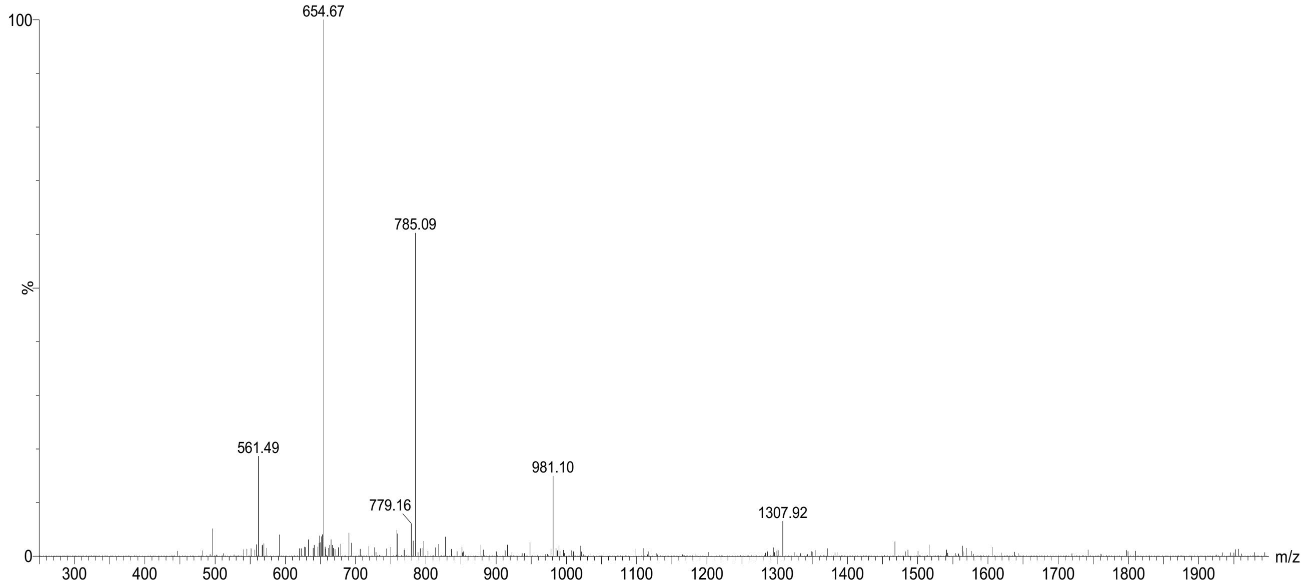

Figure S1.4*: Mass spectrum of purified peptide* ***5***

Peptide **6**

Y C Q K W M W T C D S E R K C Z E G M V Z R L W Z K K K L W **6**

Isolated yield: 13%
Scale and reagent quantities identical to Peptide **5**.

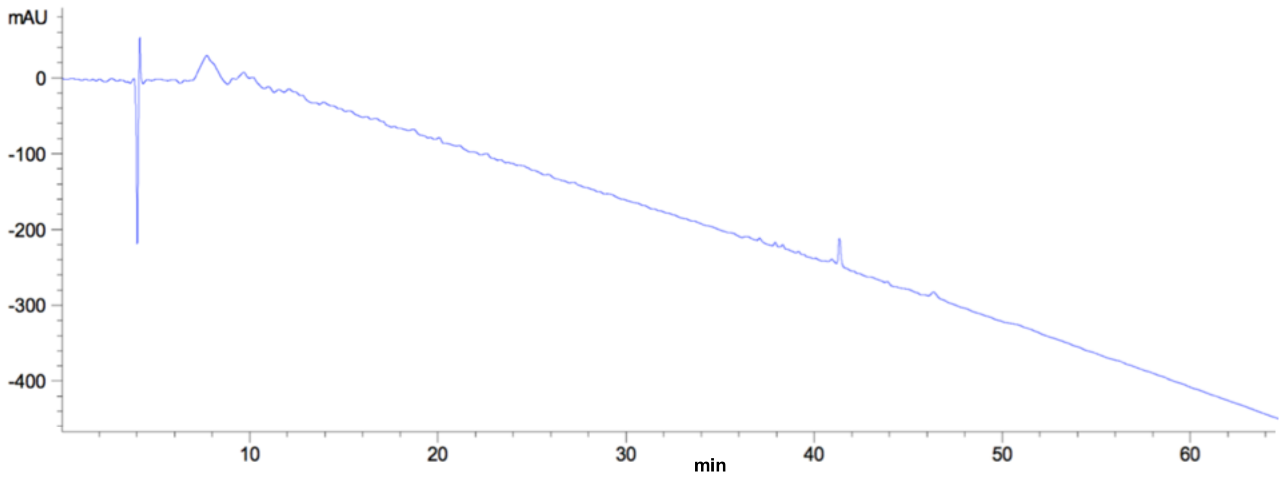

Figure S1.5**: Analytical HPLC trace for purified peptide **6

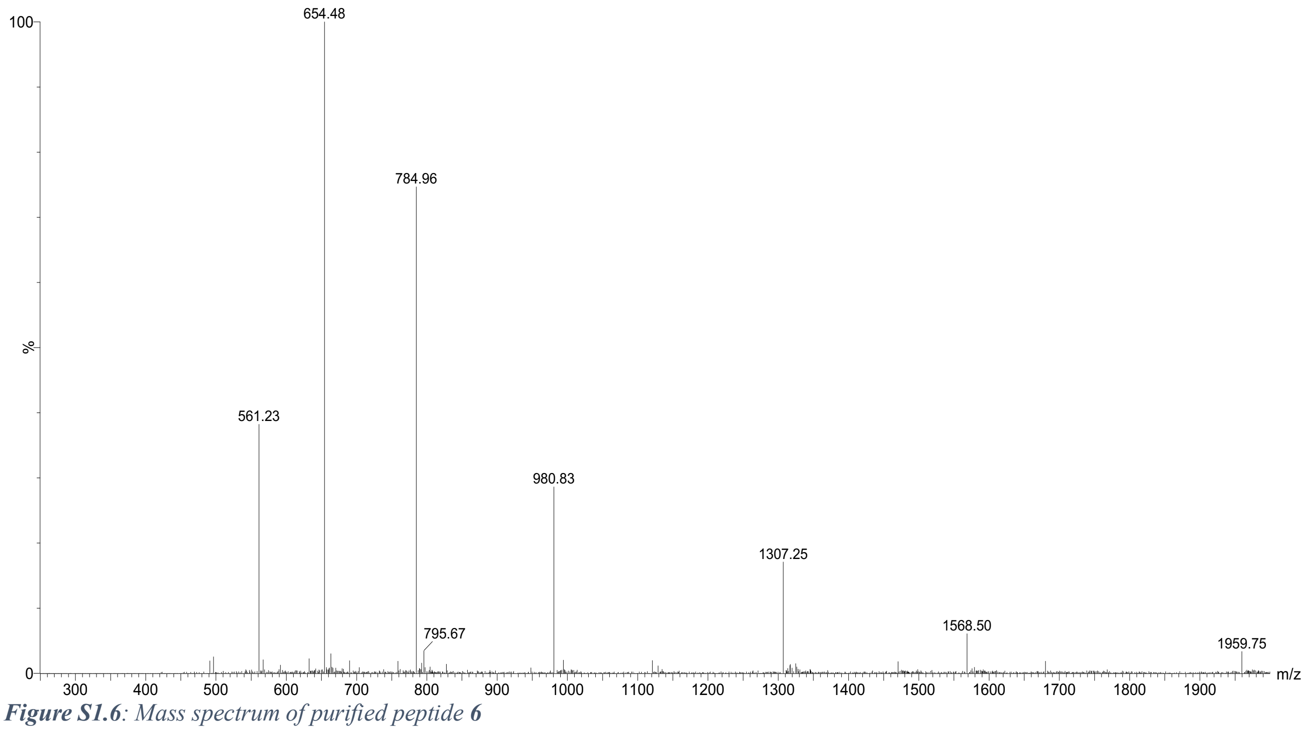

Figure S1.6**: Mass spectrum of purified peptide **6

S2 Fragmentation data for MS/MS experiments

Fragment assignments for the MS2 spectra shown in Figure 4 of the main text.

Peptide **3**

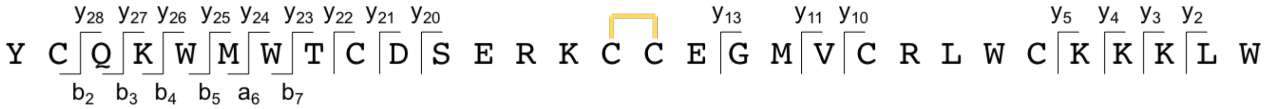

Ion	Charge	Calculated m/z	Found m/z	Error (ppm)	Intensity
b-ions					
b2	1	267.0798	267.0785	-4.87	60366
b3	1	395.1384	395.1381	-0.76	30445
b4	1	523.2334	523.2341	1.40	24472
b5	1	709.3127	709.3164	5.23	73782
	2	355.1600	355.1606	1.69	7772
b7	2	513.7199	513.7192	-1.36	3704
y-ions					
y2	1	318.1813	318.1828	4.71	120720
y3	1	446.2762	446.2788	5.73	83200
y4	1	574.3712	574.3662	-8.64	55700
y5	1	702.4662	702.4637	-3.49	4851
	2	351.7367	351.7387	5.57	3283
y10	2	682.3782	682.3848	9.73	6185
y11	2	731.9124	731.9154	4.14	39801
y13	2	825.9434	825.9441	0.91	26868
	3	550.9647	550.9647	0.05	4333
y20	3	828.7367	828.7327	-4.79	14283
	4	621.7988	621.7984	-0.68	18885
y21	3	867.0790	867.0836	5.36	105643
	4	650.5599	650.5642	6.61	12551
y22	3	901.4154	901.4113	-4.49	93007
	4	676.3122	676.3080	-6.23	53605
y23	3	935.0979	935.1011	3.35	137669
	4	701.5741	701.5757	2.28	119068
y24	3	997.1244	997.1263	1.93	76990
	4	748.0940	748.0922	-2.41	95296
y25	3	1040.8045	1040.8070	2.34	39691

	4	780.8541	780.8556	1.94	62787
y26	4	827.3739	827.3761	2.66	51211
y27	4	859.3933	859.3991	6.75	56433
	5	687.7196	687.7199	0.44	27023
y28	5	713.3264	713.3267	0.47	23950
a-ions					
a5	1	681.3178	681.3154	-3.48	14707
	2	341.1626	341.1619	-1.91	23584
a6	1	812.3583	812.3612	3.64	10372
	2	406.6828	406.6825	-0.71	8809
a7	2	499.7225	499.7235	2.10	5390

Peptide **4**

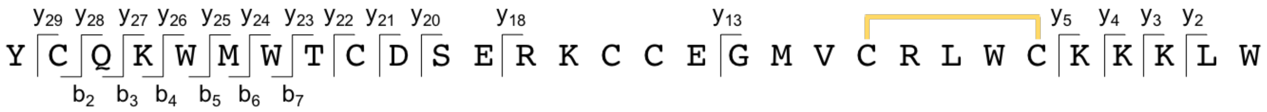

Ion	Charge	Calculated m/z	Found m/z	Error (ppm)	Intensity
b-ions					
b2	1	267.0798	267.0798	0.0000	33670
b3	1	395.1384	395.1392	2.1082	15236
b4	1	523.2334	523.2347	2.4845	13361
b5	1	709.3127	709.3146	2.6786	40538
	2	355.1600	355.1605	1.5213	3982
b6	1	840.3531	840.3589	6.8690	35911
b7	2	513.7199	513.7193	-1.0871	2496
y-ions					
y2	1	318.1813	318.1818	1.5714	51709
y3	1	446.2762	446.2764	0.4905	42545
y4	1	574.3712	574.3714	0.3482	22700
y5	1	702.4662	702.4666	0.6406	3593
y13	3	550.2927	550.2949	3.9196	5540
y18	3	756.7118	756.7093	-3.3038	2433
y20	3	828.7366	828.7378	1.4131	30899
	4	621.8043	621.8052	1.4622	10444
y21	3	867.0789	867.0739	-5.8164	65659
	4	650.5599	650.5611	1.8446	17905
y22	3	901.4154	901.4182	3.1062	66599

	4	676.3122	676.3149	3.9922	33702
y23	3	935.0979	935.1035	5.9912	85805
	4	701.5752	701.5772	2.7904	74574
y24	3	997.1244	997.1293	4.9141	38245
	4	748.0940	748.0974	4.5449	25832
y25	3	1040.8045	1040.8072	2.5973	10608
	4	780.8552	780.8567	1.9316	13965
y26	4	827.3750	827.3828	9.4032	12535
y27	4	859.3988	859.4024	4.2347	12385
	5	687.7205	687.7222	2.5245	12575
y28	5	713.3264	713.3281	2.3832	4267
y29	5	733.9340	733.9355	2.0217	55290
a-ions					
a5	1	681.3177	681.3177	-0.0416	8223
	2	341.1625	341.163	1.4586	11449
a6	1	812.3583	812.3610	3.3236	2130
	2	406.6827	406.6824	-0.8476	5175
a7	2	499.7225	499.7233	1.6009	4967

S3 Mass spectra for buffer-folded penicillamine peptides

Peptides **5** and **6** were folded using the buffer method to ensure that replacement of cysteine by penicillamine did not prevent successful folding under these conditions. Mass spectra supporting the assignment of the expected 3 disulfide bonds to the product are presented.

Fully-folded peptide **5** with 3 x S-S

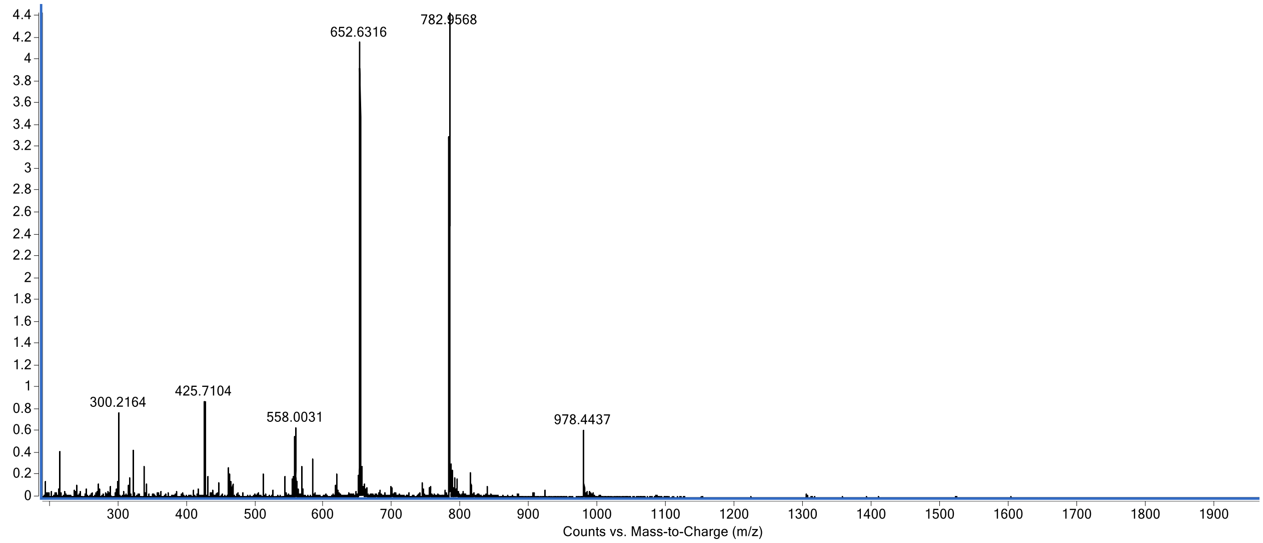

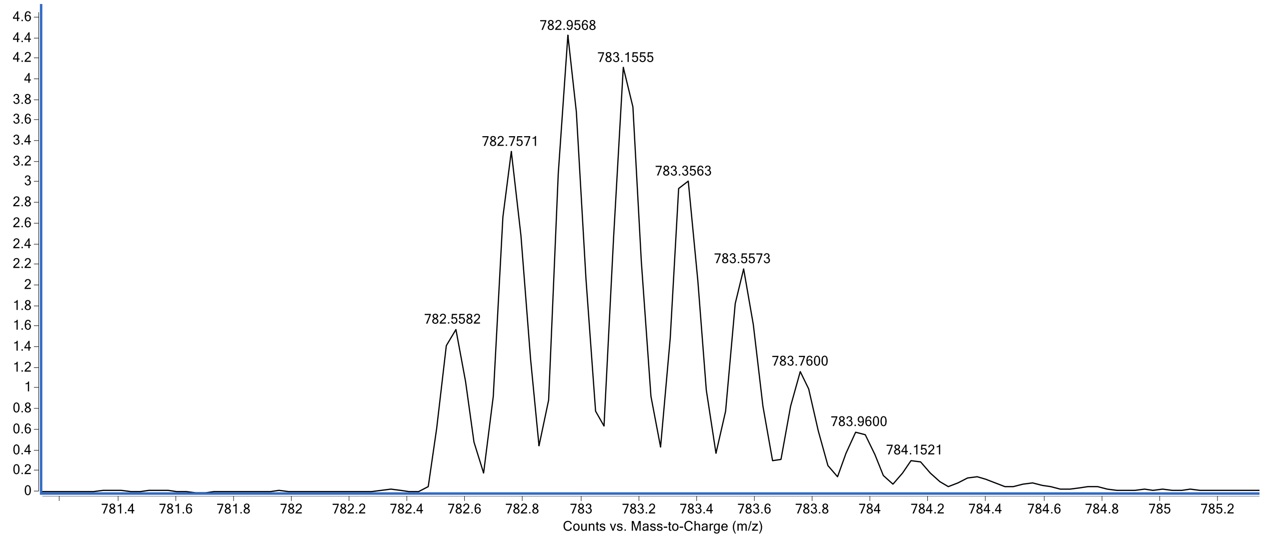

Figure S3.1: *(Top) Mass spectrum for oxidation product of peptide* ***5****, folded using the redox buffer. (Bottom) Region of the mass spectrum highlighting the +5 charge state. Calculated mass for* $[C_{174}H_{267}N_{46}O_{41}S_8]^{5+}$ *= 782.5582 m/z, found 782.5582 m/z.*

Fully-folded peptide **6** with 3 x S-S

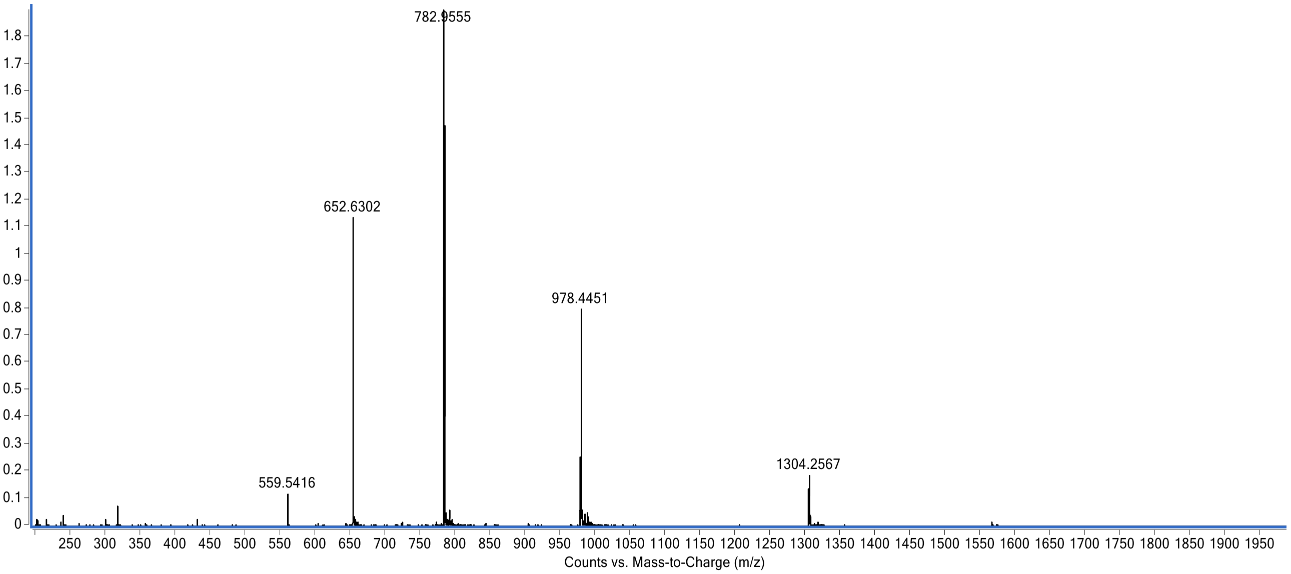

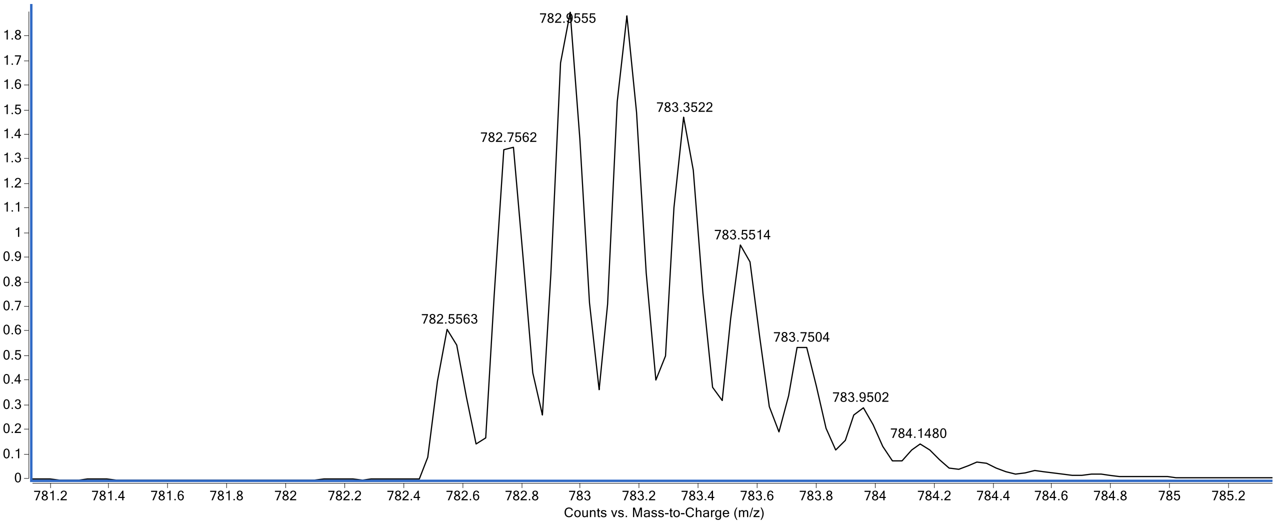

Figure S3.2*: (Top) Mass spectrum for oxidation product of peptide* ***6****, folded using the redox buffer. (Bottom) Region of the mass spectrum highlighting the +5 charge state. Calculated mass for* $[C_{174}H_{267}N_{46}O_{41}S_8]^{5+}$ *= 782.5582 m/z, found 782.5563 m/z.*

S4 Mass spectrometry of 3xS-S product from folding of peptide 6 in water

After folding of the peptides in water for 7 days, the product peaks were isolated by HPLC and the number of disulfide bonds present was determined by mass spectrometry (see main text **Figure 6**). Data is presented to support the assignment of three disulfide bonds to one product of the folding of peptide **6**.

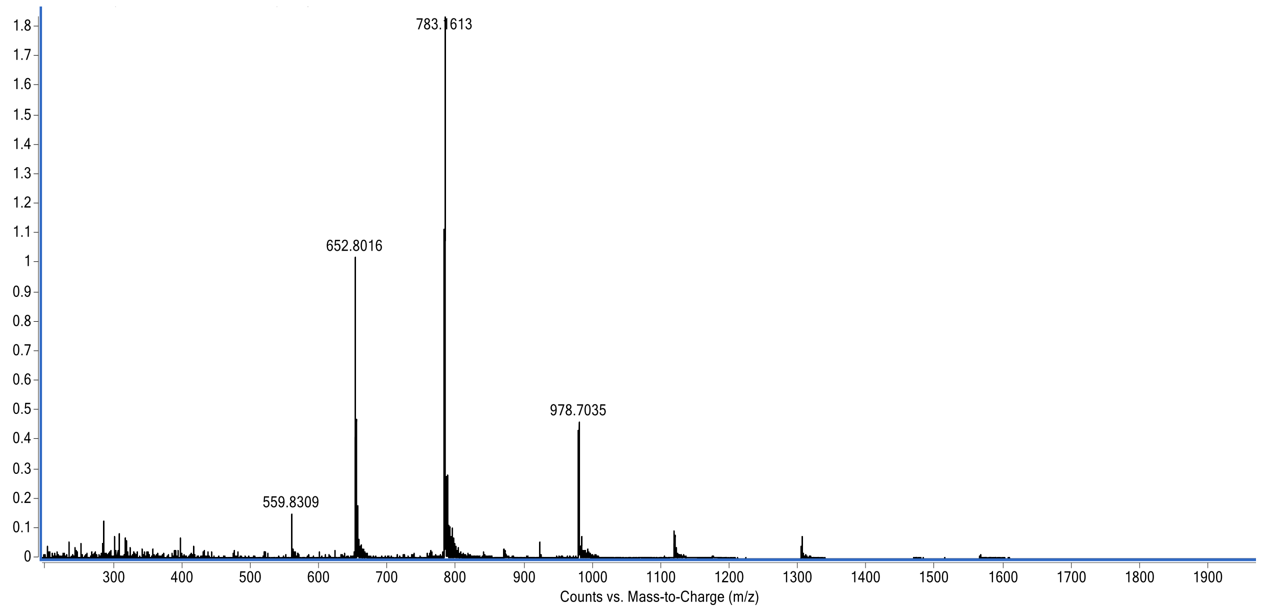

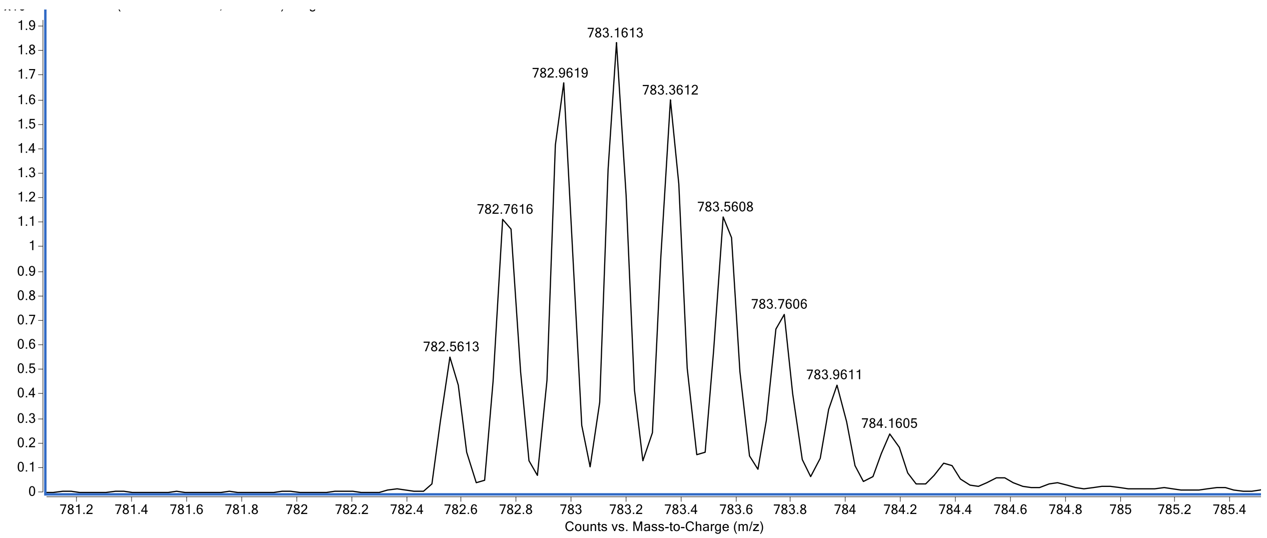

Figure S4.1*: (Top) Mass spectrum for the 3xS-S oxidation product of peptide* ***6****, folded using the water method. (Bottom) Region of the mass spectrum highlighting the +5 charge state. Calculated mass for* $[C_{174}H_{267}N_{46}O_{41}S_8]^{5+}$ *= 782.5582 m/z, found 782.5613 m/z.*

S5 Supplemental tandem mass spectra

Peptides **3** and **4** were identified by tandem mass spectrometry experiments (main text Figure 3 and Figure 4). Further peaks were observable in the chromatogram (Figure 3), but the corresponding peptides could not be fully assigned, either due to the presence of overlapping one- and two-disulfide peptides or insufficient fragmentation. The fragmentation that was observed was, however, sufficient to indicate that neither Cys(I) or Cys(II) was involved in disulfide bonding, and data to support this assertion is presented below.

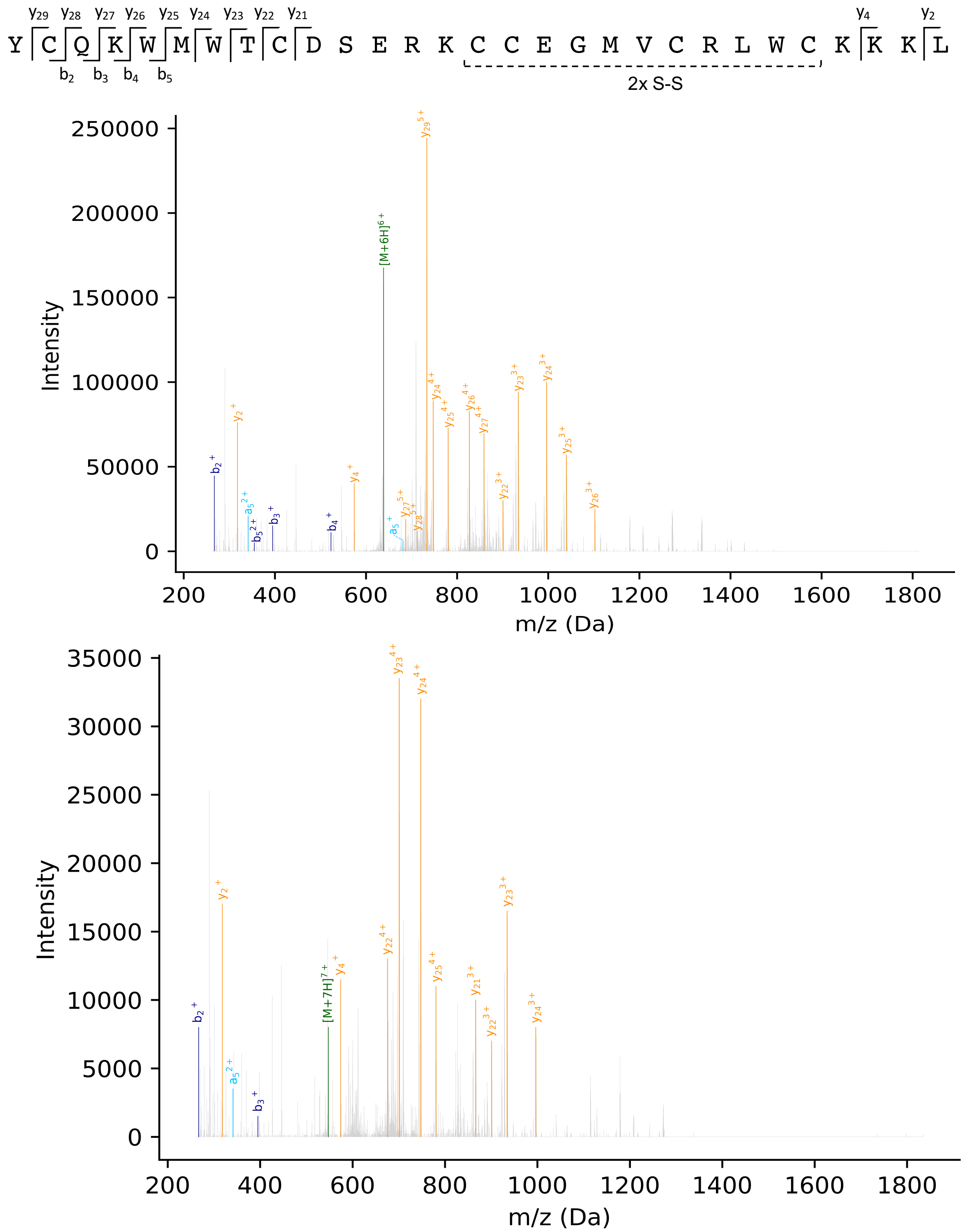

Figure S5.1: Tandem mass spectra for the two-disulfide product of folding peptide 2 in water over 7 days, showing the fragmentation of (top) the 6+ charge state and (bottom) the 7+ charge state. The fragmentation diagram shows all fragments observed. For clarity, peaks corresponding to neutral losses are not labelled.

S6 Alkylation/reduction of coeluting one- and two-disulfide peptides

To try and distinguish the coeluting peptides (orange peaks, Figure 3), the peptides were isolated by HPLC, the free cysteines were alkylated with N-ethylmaleimide (NEM), and the remaining disulfide bonds were reduced with TCEP (main text, Materials and Methods). The peptide identification protocol was performed on these samples, and the one- and two-disulfide peptides were distinguished by their different modified masses. Fragmentation was not sufficient to fully identify the disulfide bonding patterns in these peptides. Representative mass spectra are presented below.

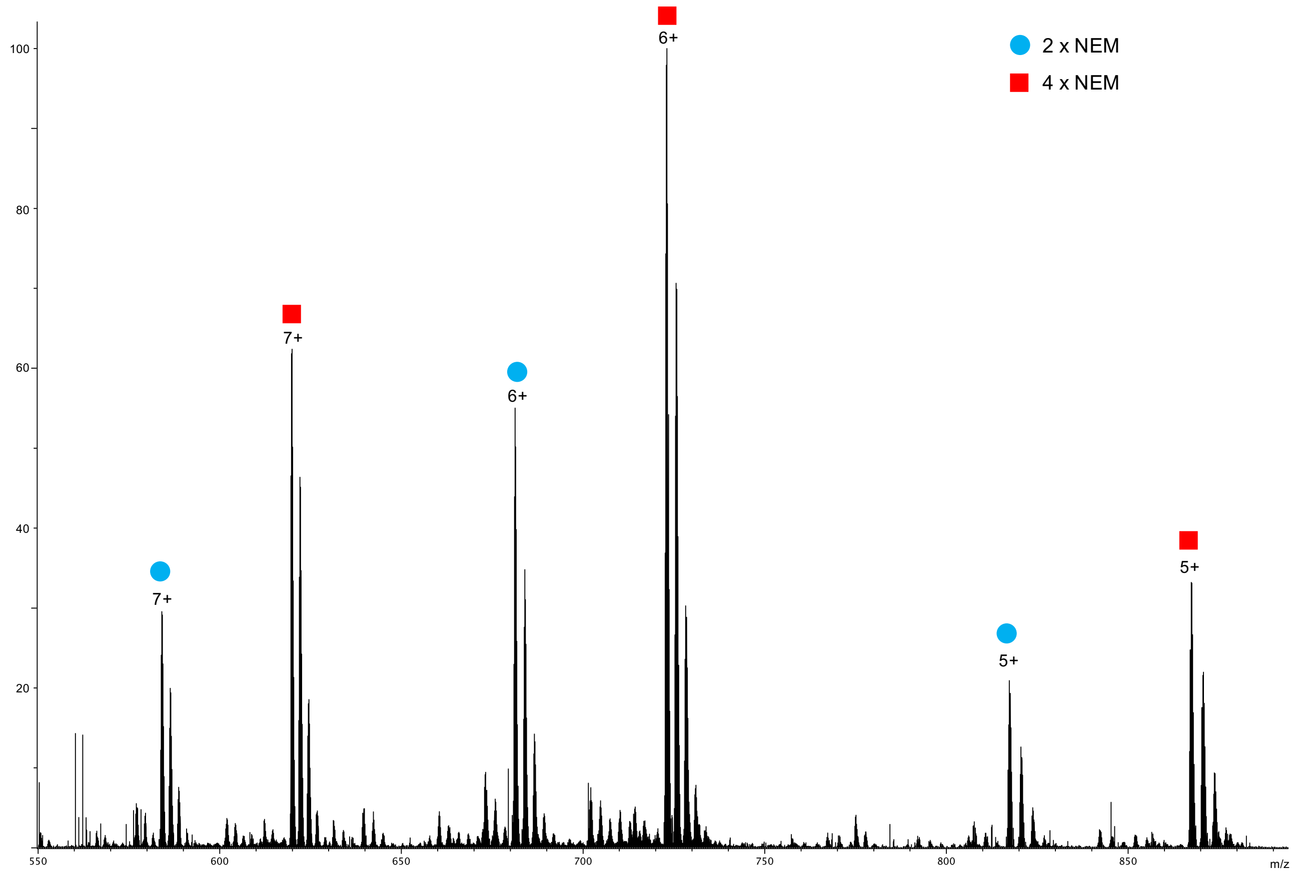

***Figure S6.1**: Representative MS1 spectrum (expanded to highlight the relevant region) of a coeluting peptide peak after alkylation and reduction. The one- and two disulfide peptides can be distinguished by their different masses (blue circles and red squares). Peaks corresponding to oxidation of methionine residues are also visible.*

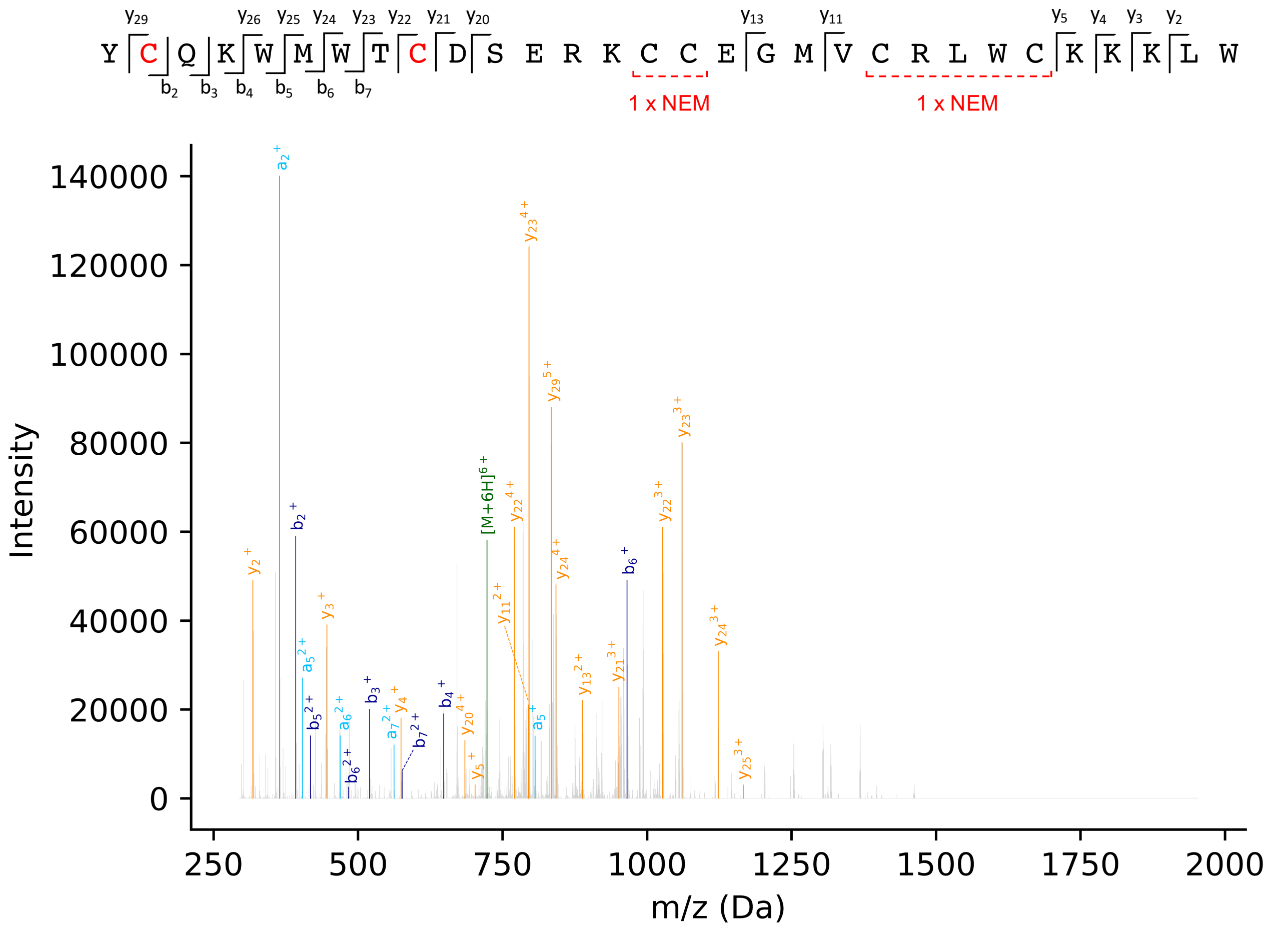

Figure S6.2: *Tandem mass spectrum of a one-disulfide peptide identified by the alkylation/reduction experiment. Cysteine residues modified by NEM are highlighted in red. NEM modifications that cannot be assigned to a cysteine due to insufficient fragmentation are labelled with red dashed brackets.*
